# Dawning aurora: TPXL3 activates α-Aurora kinase and regulates mitotic spindle morphogenesis

**DOI:** 10.1093/plcell/koaf074

**Published:** 2025-04-02

**Authors:** Renuka Kolli

**Affiliations:** Assistant Features Editor, The Plant Cell, American Society of Plant Biologists; Sainsbury Laboratory, University of Cambridge, Cambridge, UK

Aurora kinases are essential highly conserved serine/threonine kinases for the onset and progression of mitosis. The Aurora kinase family consists of Aurora-A, -B, and -C in mammals. As they are overexpressed in various human cancers, there is good therapeutic potential for inhibitors and drugs that target Aurora kinases, and numerous clinical trials are ongoing. Binding of the microtubule-associated protein TPX2 (Targeting protein for Xklp2) activates Aurora-A kinase activity and allows its association with microtubules in vertebrates (Eyers et al. 2003). The plant TPX2 ortholog contains all the conserved domains of the animal counterpart (Vos et al. 2008). Additionally, plant genomes encode several TPX2-like (TPXL) genes that lack either Xklp2-binding domain or both Aurora-binding and importin domains of TPX2 (Dvorak Tomastikova et al. 2020). *Xenopus laevis* (frog) Xklp2 is a plus-end–directed kinesin that is involved in spindle pole stability, while eukaryotic importins selectively import nuclear proteins such as TPX2. Arabidopsis TPX2 was able to restore microtubule assembly in TPX2-depleted Xenopus egg extracts, indicating functional conservation (Vos et al. 2008). However, based on genetic analyses, TPXL3, which lacks the Xklp2-binding domain but not the canonical TPX2, is essential for embryogenesis, implicating TPXL3's role in mitotic spindle morphogenesis (Boruc et al. 2019).

**Xingguang Deng and coauthors (Deng et al. 2025)** used Arabidopsis *TPXL3* repression lines to overcome the lethality from null mutation and test whether TPXL3 regulates α-Aurora (Aurora-A counterpart in plants), thereby affecting spindle morphogenesis during mitosis. An artificial microRNA construct was used to specifically target *TPXL3* mRNAs for degradation. Compared with the wild-type plants, amiR-*TPXL3* plants were growth inhibited to varying degrees that correlated with the level of mRNA reduction. After confirming that cell elongation is not affected in the mutant, the authors focused on examining cell division. Expressing GFP-fused TPXL3 under the control of the *TPXL3* native promoter in the homozygous mutant restored normal growth, and a distinct localization pattern at the mitotic spindle microtubules and the distal ends of phragmoplast microtubules facing the daughter nuclei was evident. Hence, TPXL3 could be involved in mitosis and cytokinesis.

By expressing 1 of the 2 Arabidopsis α-Aurora homologs (AUR1) fused to the FLAG peptide in the TPXL3-GFP transgenic lines and visualizing the immunofluorescence and fluorescence signals of the fusion proteins, respectively, the authors detected colocalization of AUR1-FLAG and TPXL3-GFP throughout mitosis and in the phragmoplast distal zone during cytokinesis. Then they tested whether compromised TPXL3 expression would affect AUR1 localization. While transgenic GFP-AUR1 decorated the spindle microtubules with biases toward the spindle poles in the *aur1 aur2* double mutant, it became mostly diffuse in the amiR-*TPXL3* mutant, indicating a role of TPXL3 in targeting AUR1 to spindle microtubules. The authors then went on to monitor mitotic microtubule remodeling in amiR-*TPXL3* mutant cells using a microtubule marker. In contrast to the kinetochore fibers converging toward the poles in the control cells, the microtubule arrays in the mutant did not form converged spindle poles during metaphase, and anaphase was delayed (Fig.). However, phragmoplast array formation and expansion during cytokinesis were similar to what was observed in the control cells. Expression of a microRNA-resistant form of TPXL3 in the mutant restored the typical converged spindles as well as the plant growth similar to the wild type. Hence, the authors concluded that TPXL3 is crucial for regulating mitotic spindle morphogenesis.

**Figure.**
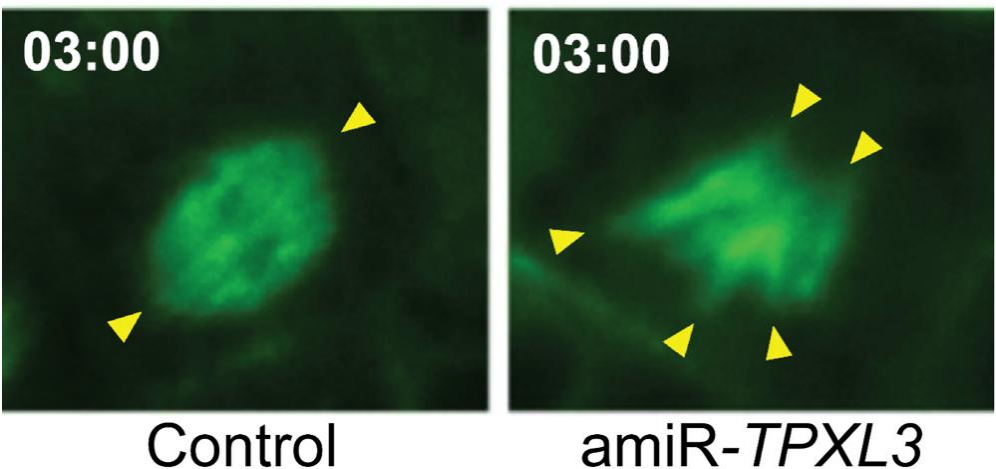
TPXL3 regulates mitotic spindle dynamics. During the metaphase at 3 min after mitosis began, spindle arrays of kinetochore fibers converged toward the poles (arrowheads) in control cells, whereas microtubule bundles point to different directions (arrowheads) in the amiR-*TPXL3* mutant. Microtubules were visualized using GFP-TUB6. Adapted from Deng et al. (2025), Figure 6.

To analyze the contribution of the different TPXL3 protein domains to its function, the authors transiently expressed truncated versions of its 5 segments fused to GFP in *Nicotiana benthamiana*. Based on the localization analyses, the authors found that AUR1 association with microtubules is dependent on domain I at the N-terminus, which contains the Aurora-binding motif. Domain II was determined to be required for microtubule binding, while domains III and IV were required for nuclear localization. The fifth domain at the C-terminus appears to be dispensable for the function of TPXL3. Furthermore, through in vitro kinase assays, the authors demonstrated that TPXL3 activates AUR1 kinase and, in turn, TPXL3 gets phosphorylated.

Thus, Deng et al. (2025) have demonstrated that Arabidopsis TPXL3 regulates α-Aurora for mitotic spindle morphogenesis by targeting it to the microtubules and activating its kinase activity. Therefore, it functions similarly to vertebrate TPX2, although the structure appears to have diverged. They found that TPXL3 also regulates the localization of the microtubule nucleating γ-tubulin complex, possibly via α-Aurora, which can be explored further in future studies. Along these lines, it is important to identify the substrates of α-Aurora kinase whose phosphorylation directly affects spindle dynamics in plants.

## Recent related articles in *The Plant Cell*

- Qian et al. (2023) showed that another TPX2-like protein, TPXL5, is involved in cortical microtubule reorganization during lateral root initiation.
- Bao et al. (2023) uncovered that 2 microtubule-associated proteins, SlMAP70 and SlIQD21a, interact to affect microtubule stability and tomato fruit shape.
- Kim et al. (2024) found that aurora kinases were downregulated in Chlamydomonas *dyrkp1* mutant exhibiting delayed parental cell wall degradation during cell division.
- Safi et al. (2023) developed a toolkit to study protein–protein interactions, phosphorylation, and other posttranslational modifications with high sensitivity.

## Data Availability

No new data were generated or analysed in support of this.

## Dive Curated Terms

The following phenotypic, genotypic, and functional terms are of significance to the work described in this paper:

TPX2 Gramene: AT1G03780
TPX2 Araport: AT1G03780

TPXL3 Gramene: At4g22860
TPXL3 Araport: At4g22860

AUR1 Gramene: At4g32830
AUR1 Araport: At4g32830
